# Health Information Exchange Usage in Japan: Content Analysis of Audit Logs

**DOI:** 10.2196/65575

**Published:** 2025-05-27

**Authors:** Jun Suzumoto, Yukiko Mori, Tomohiro Kuroda

**Affiliations:** 1 Graduate School of Medicine, Kyoto University, 43 Shogoin-kawahara-cho, Sakyo-ku, Kyoto, 606-8507, Japan, 81 753667701, 81 753667704; 2 Health Policy Bureau, Ministry of Health, Labour and Welfare, Tokyo, Japan; 3 Division of Medical Information Technology and Administration Planning, Kyoto University Hospital, Kyoto, Japan; 4 Graduate School of Informatics, Kyoto University, Kyoto, Japan

**Keywords:** health information exchange, query-based exchange, audit log, Japan, ID-Link, PicaPicaLink, Choukai Net, Asia, health information, audit, content analysis, medical data, audit logs, use, HIE, nursing

## Abstract

**Background:**

In Japan, research on the types of medical data requested by health care workers in health information exchanges (HIEs) is limited. Examining the number of views for each data type is important to quantify its benefits.

**Objective:**

This study aimed to identify the types of medical data that are frequently viewed on demand using HIEs in Japan.

**Methods:**

We analyzed audit log data from two HIEs, Choukai Net and PicaPicaLink, covering the period from April 1, 2017, to March 31, 2022. First, we calculated the cumulative monthly usage days of the HIEs by each institution for the financial year (FY) 2021/22. Second, we calculated the cumulative annual usage days of the HIEs by each user type for FY 2021/22. Third, we calculated the view rate for each output field and content within each HIE, using institution type or year as the aggregation unit. Fourth, we calculated the cumulative annual usage days of the HIEs for days with and without progress note viewing, and for days without any content viewing. Fifth, we calculated the cumulative number of viewed days for content scheduled to be included in the national HIE compared to that which was not.

**Results:**

In 32.6% (47/144) of hospitals connected to Choukai Net and 2.3% (20/875) of hospitals connected to PicaPicaLink, cumulative monthly usage days exceeded 101. Additionally, in 32.4% (56/173) of visiting nursing stations connected to Choukai Net, cumulative monthly usage days were over 51. User types viewing HIEs were heavily biased toward institution types other than hospitals. The overall view rate for progress notes was highest among all content types, at 67.4% (83,476/123,915) for Choukai Net and 32.9% (26,159/79,612) for PicaPicaLink. In both HIEs, when comparing by institution type, the view rate for progress notes was highest for visiting nursing stations, reaching 91.8% (5553/6052) for Choukai Net and 65.3% (126/193) for PicaPicaLink. We also found that 17% (5417/31,944) of Choukai Net usage and 9.6% (1802/18,862) of PicaPicaLink usage involved referencing only progress notes in FY 2021/22. The view rate of content scheduled to be included in the national HIE was 45.6% (56,499/123,791) for Choukai Net and 47.7% (37,972/79,612) for PicaPicaLink. Conversely, the view rate for content not scheduled to be included in the national HIE was higher, at 80.2% (99,234/123,791) for Choukai Net and 56.6% (45,052/79,612) for PicaPicaLink.

**Conclusions:**

In both HIEs analyzed in this study, progress notes were the most viewed content. As more health care organizations disclose the progress notes they manage to their HIEs, progress notes are likely to be viewed more frequently. The cost-benefit of disclosing progress notes to HIEs remains unclear, and both health care providers and patients have concerns about privacy risks. Future research is needed to quantify and maximize the benefits of disclosure while mitigating the associated privacy risks.

## Introduction

Recently, health care delivery systems have undergone a profound global transformation catalyzed by advancements in information technology. Among the numerous innovations, the health information exchange (HIE) stands out as a remarkable tool for revolutionizing the health care sector by facilitating electronic data sharing among health care providers [1]. Currently, at least 200 HIEs are operating in Japan [2]. The most common feature of an HIE in Japan is on-demand viewing or searching for medical data from other institutions [3,4], also known as a query-based exchange or query-based HIE [1,5,6]. In addition to already established regional HIEs, the Ministry of Health, Labour and Welfare (MHLW) is considering the establishment of a nationwide HIE. The MHLW intends to share 3 types of documents and 6 types of information (3D6I) [7] across this nationwide HIE, which is referred to as Clinical Information Sharing (CLINS) [8].

It is crucial to investigate the types of medical data required by health care workers using the HIE, as medical data, which are in high demand, may not always be disclosed to Japanese HIEs. In Japan, the types of medical data disclosed to HIEs vary among HIEs. For example, a 2023 questionnaire survey targeting 48 HIEs revealed that while prescription data and laboratory test data were disclosed in all HIEs, the disclosure rate of progress notes written by doctors was only 41% [9]. This variation in data disclosure is further complicated by the differences in the data types provided by each institution to the HIE. Sharing data incurs costs, making it crucial to assess the demand and value of each data type and share it selectively.

In Japan, research on the types of medical data in demand for HIEs is limited. The MHLW conducted at least 2 studies on the types of data that should be included in the CLINS [10,11]. However, both surveys were based on interviews or questionnaires with medical workers and did not incorporate data from regional HIEs. As noted above, not all data types are available in existing HIEs; however, analyzing the audit logs of HIEs to clarify the data types can provide valuable quantitative data.

To address these data type clarity limitations, this study aimed to determine the types of data frequently viewed on query-based exchanges in Japan by analyzing HIE audit logs. Considering our internal analysis of audit logs in a previous study [4], we suspected that there may be a large demand for progress notes written by doctors. Therefore, we analyzed audit logs of HIEs that share progress notes in this study.

## Methods

### HIE for Data Analysis

#### Selection of HIEs

We collected data on HIEs that met the following inclusion criteria: (1) they were included in the survey report “About the current situation of regional healthcare network” published by the MHLW, (2) the HIE was connected to more than 100 institutions and had more than 10,000 patients according to the report, and (3) progress notes from at least some of the connected institutions were shared in the HIE as of March 2022. The first two criteria were included in previous studies, while the third criterion was new to this study. We approached operators of HIEs that met these criteria to participate in the study and obtained consent from the operators of 2 HIEs, as described below.

#### Overview of Regions and HIEs

The first HIE, Choukai Net, operates in the Shonai medical area of Yamagata Prefecture [12]. The second HIE, PicaPicaLink, operates in Saga Prefecture [13].

These HIEs are operated by different regional health information organizations—the Shonai Medical Information Network Council and the Saga Prefecture Medical Information Regional Collaboration System Council, respectively. Both HIEs use the same commercial HIE, ID-Link [14], developed by SEC Co Ltd, with NEC Corporation as the service contact. ID-Link is a computer system that enables viewing patient data stored in other institutions using a query-based exchange. Institutions using ID-Link are categorized as either data disclosure institutions (DDIs), which disclose their patient data to the HIE, or data viewing institutions (DVIs), which only view the patient data. The DDIs can also view the patient data disclosed to the HIE. Table 1 presents the basic statistics for both HIEs and the regions in which they operate. Choukai Net collaborates with medical institutions from other HIEs; however, these institutions are not included in this analysis. The population in both regions is declining, with both regions experiencing a stronger trend of population decline than the national average and aging populations. In addition, the Shonai medical area, where Choukai Net operates, has a population density about one-third of the national average.

The type of disclosed patient data differed by DDI. Tables S1 and S2 in Multimedia Appendix 1 list the DDIs and the types of disclosed patient data. Additionally, there are restrictions on the types of jobs that use HIE. Choukai Net is available only to medical personnel and nursing care support specialists who are required by law or contracts (or labor contracts among DVI employees) to maintain confidentiality [15]. For employees who belong to DVIs other than nursing care institutions, occupations that can use PicaPicaLink are determined by the respective institution’s manager. For DVI employees in nursing care institutions, the occupations that can use PicaPicaLink are limited to those with national qualifications that are legally required to maintain confidentiality [16].

**Table 1.** Basic information on regions and health information exchanges.

Characteristics		Choukai Net	PicaPicaLink
Estimated area population, n^a^		257,122	800,668
Population density of the area, people/km^2b^		109.50	332.50
Proportion of population aged ≥65 years in the region^c^, %		36.10	30.60
Regional population growth rate from 2015 to 2020^d^, %		−5.76	−2.57
Number of participating patients, n^a^		55,131	559,650
**Number of data viewing institutions, n** ^**a**^			
	Hospital	12^e^	73
	Medical clinic	78	154
	Dental clinic	22	8
	Pharmacy	25	125
	Visiting nursing station	14	10
	Nursing facilities	83	22
Number of data disclosure institutions, n^a^		7	15
Year in which the health information exchange started operation		2011	2010

a Aggregated values as of the end of March 2022.

b Census data from the Ministry of Internal Affairs and Communications in 2020. The national average is 338.2 people/km2.

c Census data from the Ministry of Internal Affairs and Communications in 2020. The national average is 28.6%.

d Census data from the Ministry of Internal Affairs and Communications in 2015 and 2020. The national average is –0.75%.

e Yamagata University Hospital, Yamagata Prefectural Central Hospital, Yamagata Prefectural Shinjo Hospital, Okitama Public General Hospital, and Sanyudo Hospital are excluded from the tally.

#### Patient Consent

Both HIEs require explicit patient consent before health care workers can use the HIE to view a patient’s medical data [17,18]. If a DVI medical worker wants to use the HIE to refer to a certain patient’s medical data stored in the DDI (eg, for treatment purposes), the medical worker must first explain the HIE to the patient. If the patient agrees to participate, they must complete a written consent form providing their demographic data, such as their name and date of birth. The DVI health care worker then adds the name of the DDI requesting disclosure of the patient’s medical data and faxes the consent form. For Choukai Net, the consent form is sent to the HIE secretariat, and for PicaPicaLink, it is sent to the DDI requesting the disclosure. At the institution receiving the fax, an administrative worker releases the patient’s medical data, as specified in the consent form, from the designated DDI to the fax sender’s DVI. This process allows medical workers at the DVI to view the patient’s medical data stored in the DDI using the HIE. Other DVIs cannot view the patient’s medical data stored in the DDI. Once a patient consents to the disclosure of their medical data from the DDI to the DVI and the process is completed, the patient does not need to complete the consent form again for the same institution. When disclosing patient medical data from another DDI or to a new DVI, the patients must fill out an additional consent form.

However, there are some exceptions to the above operation. The first exception is when medical workers use the “emergency medical service” (EMS) [19]. The EMS is the default feature of ID-Link. Typically, DVI health care workers cannot view a patient’s medical data unless the DDI actively discloses the patient’s data. However, this feature allows DVI health care workers to view patient data without waiting for the DDI to take action. When DVI health care workers urgently need to view a patient’s medical data stored in a DDI, they can do so by entering the patient’s ID from the DDI into the HIE in a certain way. Medical workers can use the EMS in 2 main situations. The first situation is when emergency medical care is required, and the patient is unable to fill out a consent form. The second case is when medical data need to be accessed, but the DDI or HIE office is unable to perform operations for data disclosure.

The second exception is the “patient demographic data synchronization feature” (PDDSF) [20] and “name matching feature” (NMF) [21]. These features are available only in PicaPicaLink and are not available in Choukai Net. PDDSF allows for all basic patient data stored in DDIs to be uploaded to the ID-Link Data Center. These basic data include the name, date of birth, gender, address, telephone number, and insurance number of a patient. The NMF enumerates the IDs of the same patient in each DDI by comparing patient data collected using the PDDSF, thus enabling name matching. These features are used when DVI medical workers need to access a patient’s medical data stored in a specific DDI using the EMS function but do not know the patient’s ID in the DDI.

The third exception is an interagency data-sharing feature called “Sagan-Path Net” [22]. This feature is also available on PicaPicaLink and allows facilities to share summary data of stroke patients transferred from acute care hospitals to rehabilitation hospitals. To use this feature for a patient, the patient must also fill out a consent form, but they cannot specify the DVI details for each DDI. Instead, all DVIs with access to the patient’s data through PicaPicaLink can view the shared summary data using Sagan-Path Net.

#### Flow of HIE Use

**Figure 1.**
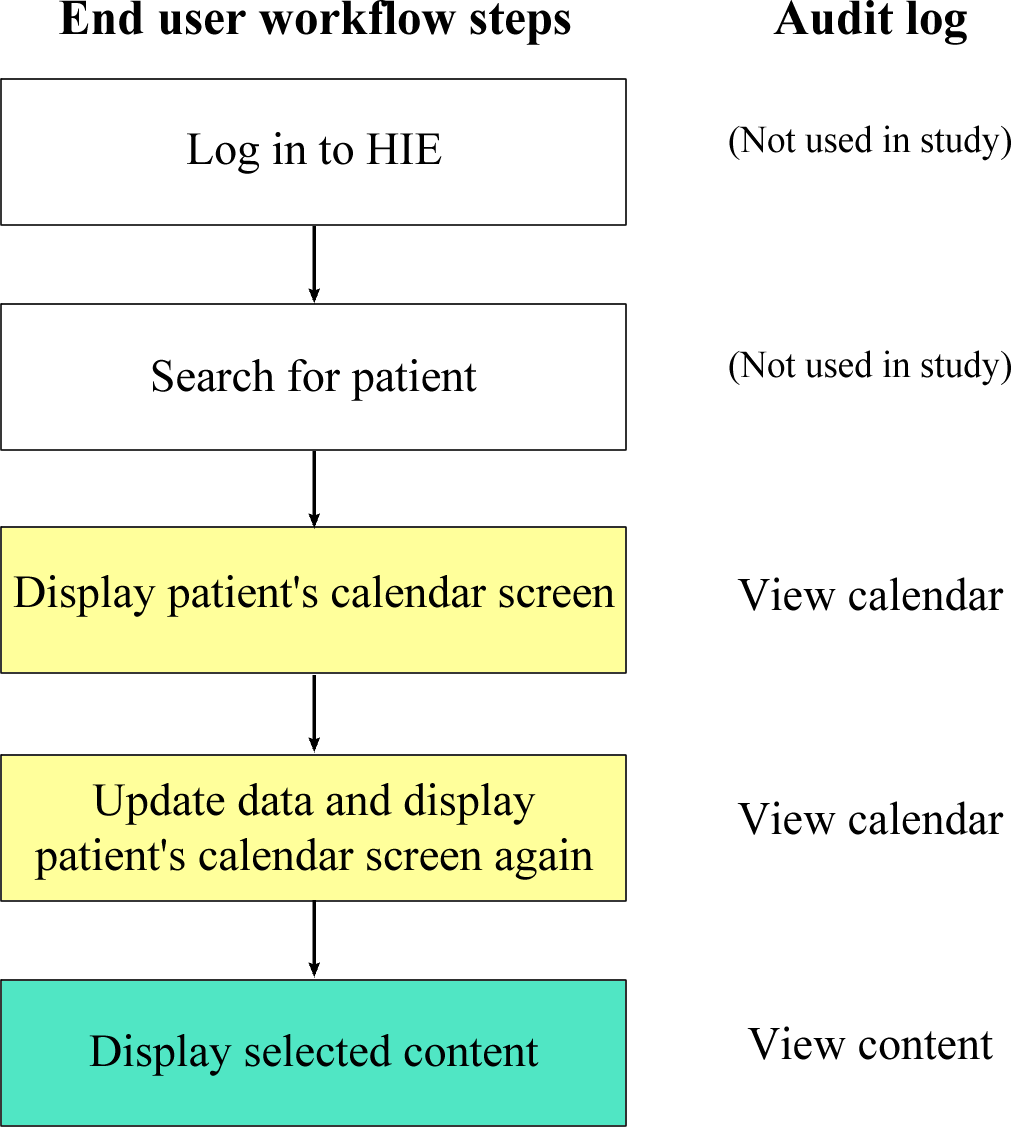
Workflow when end users use ID-Link. We analyzed the usage data of users who had at least accessed the patient’s calendar screen. HIE: health information exchange.

As explained in the previous section, both HIEs require explicit patient consent to view their data, except in emergencies by using the EMS functionality. A user logs in to the HIE with their ID and password. Subsequently, the user enters the name or registration ID of the patient whose information they want to view in the search field. Patients viewed within the past 14 days are displayed in a list, allowing the user to select from the list. When the desired patient appears in the list, the user clicks to select the patient option. The selected patient’s medical information is then displayed in chronological order by item, known as the patient’s calendar screen. When the user first opens the patient’s calendar screen, the data remain unchanged from the last time they viewed the patient’s page. Therefore, it is necessary to press the data-update button to obtain the most recent patient data. Once the data are updated, users can review the patient’s calendar screen and select the contents displayed. This series of steps is illustrated in Figure 1.

### Ethical Considerations

This study was approved by the ethics committee of Kyoto University Graduate School and Faculty of Medicine (accession number R3266-7). The disclosure document outlining the research plan and data to be extracted were published on the Kyoto University Hospital website [23], ensuring that research subjects had the opportunity to opt out. Personal data obtained in the study were pseudonymized by the HIEs that provided the data. Research subjects did not receive compensation.

### Data Collection

We collected profile and audit log data from health care workers enrolled in the 2 HIEs. The type of data collected was consistent with that from our previous study [4]. The data that were collected are presented in Textbox 1.

The data extraction period was from April 1, 2017, to March 31, 2022. We also obtained data on the number of participating institutions and patients per month for each HIE. The IDs of the patients accessed by medical workers were not extracted in this study.

Textbox 1. **Health information exchange user data collected in this study.**

- Occupation Institution
- Anonymous identifier
- Date of account registration and account deletion in health information exchange
- Date and time of access to health information exchange
- Type of data accessed by the user
- Access details: calendar access or content access? If content access, what content?

### Measures and Data Analyses

#### Overview

For each user, we counted records of “View calendar” or “View content” in the audit log, as shown in Figure 1, as a measure of HIE use. The aggregation unit used was days. When counting the number of views for a specific type of output field or content, we extracted only the relevant audit logs and counted them in the same way. As a unit for counting HIE use by group, 1 man-day was defined as HIE use on 1 day with 1 user account. The cumulative number of accesses to an institution was calculated as the total number of days of HIE use by each user associated with that institution.

We classified the institutions enrolled in HIE into 6 categories: hospitals, medical clinics, dental clinics, pharmacies, visiting nursing stations, and nursing facilities. Institutions that could not be classified into any of these categories were excluded from the analysis. For example, this study did not analyze public institutions, such as fire departments, public health centers, local medical associations, or HIE development vendors.

The financial year (FY) ran from April 1 of one year to March 31 of the following year. For example, FY 2021/22 started on April 1, 2021, and ended on March 31, 2022. R software (version 4.3.1; R Foundation for Statistical Computing) was used for the analysis.

#### Man-Days for Monthly HIE Use by Each Institution in FY 2021/22

We calculated the man-days for monthly HIE use by each institution and aggregated them by institution type. For each institution enrolled in both HIEs, the man-days for monthly HIE use in FY 2021/22 were aggregated. Subsequently, for each institution type, we tallied the number of months for each man-day group, which was divided into 5- or 10-day increments. Finally, the percentage of each man-day group was calculated for each institution type. This analytical method is consistent with that used in our previous study [4].

#### Man-Days for Annual HIE Use by User Type and Institution Type in FY 2021/22

We classified all user occupation data into 8 user types: “doctor,” “nurse,” “rehabilitation staff,” “pharmacist,” “dental profession,” “nursing care staff,” “other medical professions,” and “type unknown.” For each institution category, we aggregated the total number of man-days of HIE use by user type in FY 2021/22.

#### Man-Days and View Rate for Each Output Field and Content

For each HIE, we calculated the man-days and view rate for each output field and content. The aggregation units were FY and institution type. In the analysis using FY as the aggregation unit, all log data from the 5-year period were analyzed. In the analysis using institution type as the aggregation unit, only audit log data for FY 2021/22 were analyzed. The viewed output fields and content were listed for each man-day of HIE use. We then tallied the total number of man-days for HIE use, the number of man-days for views of each output field, and the content in each aggregation unit. Finally, we calculated the view rate for each output field and content in each aggregation unit. The view rates of the output field and content are defined as follows:


(1) $$\begin{array}{c} \frac{Number\;of\;man‐days\;for\;viewing\;the\;output\;field\;or\;content\;in\;the\;aggregation\;unit}{Total\;number\;of\;man‐days\;of\;HIE\;use\;in\;the\;aggregation\;unit}\times 100. \end{array}$$

In the analysis using FY as the aggregation unit, we presented the top 5 content views by man-days over the total data analysis period in bar graphs. In the analysis using institution type as the aggregation unit, we listed the man-days and view rates for each output field and content.

As a supplementary analysis, we divided all man-days into 2 categories: those that included audit logs of viewing any content and those that only included audit logs of viewing the patient’s calendar screen (Figure 1). Man-days that included audit logs of viewing any content were further categorized as follows: man-days where (1) the only viewed content was progress notes, (2) the viewed content included both progress notes and other content, and (3) the viewed content did not include progress notes. This analysis was conducted to evaluate the impact of the disclosure of progress notes on usage man-days.

#### Man-Days of Views of Content That Corresponds to 3D6I and Content That Does Not Correspond to 3D6I

For each HIE and FY, we calculated the number of man-days spent viewing content that corresponded to 3D6I as well as content that did not correspond to 3D6I. The 3 types of documents in 3D6I were patient referral documents, discharge summaries, and health examination documents. The 6 data types in 3D6I are as follows: the name of the illness or injury; infectious disease; drug contraindications; allergies; laboratory test results and prescriptions written in the patient referral document; and discharge summary.

First, the total number of man-days for each HIE used in each FY was tallied. Subsequently, we sorted all content into categories corresponding to 3D6I and those that did not. The results for each content type are listed in Table S3 in Multimedia Appendix 2. We then tallied the number of man-days during which any content corresponding to 3D6I was viewed for each FY or HIE. We also tallied the number of man-days spent viewing content that did not correspond to 3D6I for both FY and HIE. Finally, we calculated the percentage of man-days in which any content corresponding to 3D6I was viewed, as well as the percentage of man-days in which any content not corresponding to 3D6I was viewed.

## Results

### Man-Days for Monthly HIE Use by Each Institution in FY 2021/22

Table 2 presents man-days of monthly HIE use in hospitals. For hospitals, the percentage of man-days with monthly HIE use of 0 was 9% in this study, which is less than one-fifth of that of our previous study [4]. Similarly, the percentage of man-days with monthly HIE use of 101 or more was 32.6%, which is more than 10 times that in our previous study. PicaPicaLink usage closely aligned with that reported in our previous study.

Table 3 presents man-days of monthly HIE use in other institution types. Choukai Net was actively used in some visiting nursing stations. The percentage of man-days with monthly HIE use of 26 or more was 42.2%, which is approximately 6 times that of our previous study [4]. Such high frequency of usage by visiting nursing stations was not observed on PicaPicaLink. Overall, HIE use by dental clinics was low, consistent with our previous findings [4].

**Table 2.** Distribution of man-days for monthly health information exchange, use of hospitals in financial year 2021/22.

Man-days for monthly health information exchange, use per institution, n	Choukai Net, n (%)	PicaPicaLink, n (%)
0	13 (9)	430 (49.1)
1‐10	31 (21.5)	217 (24.8)
11‐20	19 (13.2)	99 (11.3)
21‐30	9 (6.3)	50 (5.7)
31‐40	1 (0.7)	25 (2.9)
41‐50	3 (2.1)	15 (1.7)
51‐60	4 (2.8)	13 (1.5)
61‐70	4 (2.8)	3 (0.3)
71‐80	5 (3.5)	0 (0)
81‐90	6 (4.2)	0 (0)
91‐100	2 (1.4)	3 (0.3)
≥101	47 (32.6)	20 (2.3)
Total	144 (100)	875 (100)

**Table 3.** Distribution of man-days for monthly health information exchange use in other institution types in financial year 2021/22.

Man-days for monthly health information exchange use, n		Medical clinic, number of months (%)	Dental clinic, number of months (%)	Pharmacy, number of months (%)	Visiting nursing station, number of months (%)	Nursing facility, number of months (%)
**Choukai Net**						
	0	418 (44.3)	251 (95.1)	216 (72)	43 (24.9)	612 (62.6)
	1‐5	246 (26.1)	13 (4.9)	54 (18)	12 (6.9)	117 (12)
	6‐10	66 (7)	0 (0)	10 (3.3)	9 (5.2)	59 (6)
	11‐15	37 (3.9)	0 (0)	5 (1.7)	16 (9.2)	45 (4.6)
	16‐20	30 (3.2)	0 (0)	12 (4)	17 (9.8)	40 (4.1)
	21‐25	74 (7.8)	0 (0)	3 (1)	3 (1.7)	45 (4.6)
	26‐30	37 (3.9)	0 (0)	0 (0)	1 (0.6)	20 (2)
	31‐35	14 (1.5)	0 (0)	0 (0)	2 (1.2)	12 (1.2)
	36‐40	9 (1)	0 (0)	0 (0)	2 (1.2)	13 (1.3)
	41‐45	3 (0.3)	0 (0)	0 (0)	4 (2.3)	4 (0.4)
	46‐50	1 (0.1)	0 (0)	0 (0)	8 (4.6)	4 (0.4)
	≥51	9 (1)	0 (0)	0 (0)	56 (32.4)	7 (0.7)
	Total	944 (100)	264 (100)	300 (100)	173 (100)	978 (100)
**PicaPicaLink**						
	0	1303 (71)	96 (100)	1284 (85.1)	93 (77.5)	154 (60.9)
	15	219 (11.9)	0 (0)	120 (8)	11 (9.2)	33 (13)
	6‐10	85 (4.6)	0 (0)	38 (2.5)	8 (6.7)	12 (4.7)
	11‐15	80 (4.4)	0 (0)	16 (1.1)	8 (6.7)	7 (2.8)
	16‐20	77 (4.2)	0 (0)	23 (1.5)	0 (0)	5 (2)
	21‐25	47 (2.6)	0 (0)	7 (0.5)	0 (0)	5 (2)
	26‐30	10 (0.5)	0 (0)	8 (0.5)	0 (0)	8 (3.2)
	31‐35	5 (0.3)	0 (0)	9 (0.6)	0 (0)	5 (2)
	36‐40	3 (0.2)	0 (0)	2 (0.1)	0 (0)	6 (2.4)
	41‐45	3 (0.2)	0 (0)	1 (0.1)	0 (0)	4 (1.6)
	46‐50	2 (0.1)	0 (0)	0 (0)	0 (0)	2 (0.8)
	≥51	0 (0)	0 (0)	0 (0)	0 (0)	12 (4.7)
	Total	1834 (100)	96 (100)	1508 (100)	120 (100)	253 (100)

### Man-Days for Annual HIE Use by User Type and Institution Type in FY 2021/22

Table 4 presents man-days of annual HIE use by user type and institution type in both HIEs in FY 2021/22. In both HIEs, there are significant differences in the proportion of user types depending on the institution type. For hospitals, no single occupation accounts for half of the man-days. On the other hand, doctors at medical clinics, dentists at dental clinics, pharmacists at pharmacies, nurses at visiting nursing stations, and nursing care staff at nursing homes each account for more than half of the total man-days for their respective institution type. These distributions reflect the characteristics of each institution type.

**Table 4.** Man-days of annual health information exchange use by user type and institution type in financial year 2021/22.

Health information exchange and user type		Hospital, n (%)	Medical clinic, n (%)	Dental clinic, n (%)	Pharmacy, n (%)	Visiting nursing station, n (%)	Nursing facility, n (%)
**Choukai Net**							
	Doctor	5906 (42.6)	5054 (80.1)	0 (0)	0 (0)	0 (0)	516 (9.8)
	Nurse	2357 (17)	845 (13.4)	0 (0)	0 (0)	5644 (93.3)	680 (12.9)
	Rehabilitation staff	860 (6.2)	0 (0)	0 (0)	0 (0)	144 (2.4)	1 (0)
	Pharmacist	906 (6.5)	0 (0)	0 (0)	555 (97.7)	0 (0)	0 (0)
	Dental professional	4 (0)	0 (0)	13 (68.4)	0 (0)	0 (0)	0 (0)
	Nursing care staff	219 (1.6)	0 (0)	0 (0)	0 (0)	20 (0.3)	3929 (74.5)
	Other medical professionals	3263 (23.5)	335 (5.3)	6 (31.6)	13 (2.3)	60 (1)	100 (1.9)
	Type unknown	344 (2.5)	75 (1.2)	0 (0)	0 (0)	184 (3)	51 (1)
	Total	13,859 (100)	6309 (100)	19 (100)	568 (100)	6052 (100)	5277 (100)
**PicaPicaLink**							
	Doctor	2416 (27.2)	3812 (70.2)	0 (0)	254 (13.1)	0 (0)	92 (4.1)
	Nurse	640 (7.2)	400 (7.4)	0 (0)	0 (0)	192 (99.5)	602 (26.9)
	Rehabilitation staff	202 (2.3)	9 (0.2)	0 (0)	0 (0)	0 (0)	71 (3.2)
	Pharmacist	146 (1.6)	0 (0)	0 (0)	1641 (84.5)	0 (0)	0 (0)
	Dental professional	7 (0.1)	0 (0)	0 (0)	0 (0)	0 (0)	5 (0.2)
	Nursing care staff	1191 (13.4)	0 (0)	0 (0)	0 (0)	0 (0)	1152 (51.6)
	Other medical professionals	3686 (41.5)	839 (15.4)	0 (0)	35 (1.8)	0 (0)	308 (13.8)
	Type unknown	590 (6.6)	374 (6.9)	0 (0)	11 (0.6)	1 (0.5)	4 (0.2)
	Total	8878 (100)	5434 (1000)	0 (0)	1941 (100)	193 (100)	2234 (100)

### Man-Days and View Rate for Each Output Field and Content

#### Man-Days for Content View

In both HIEs analyzed, the most viewed data were progress notes. On Choukai Net, the overall number of man-days for progress notes was 83,476 and the overall view rate for progress notes was 67.4% (83,476/123,915). This was nearly 3 times higher than the overall man-days for patient summary, which was the second most viewed content. On PicaPicaLink, the number of overall man-days for progress notes was 26,159, and the overall view rate for progress notes was 32.9% (26,159/79,612).

**Figure 2.**
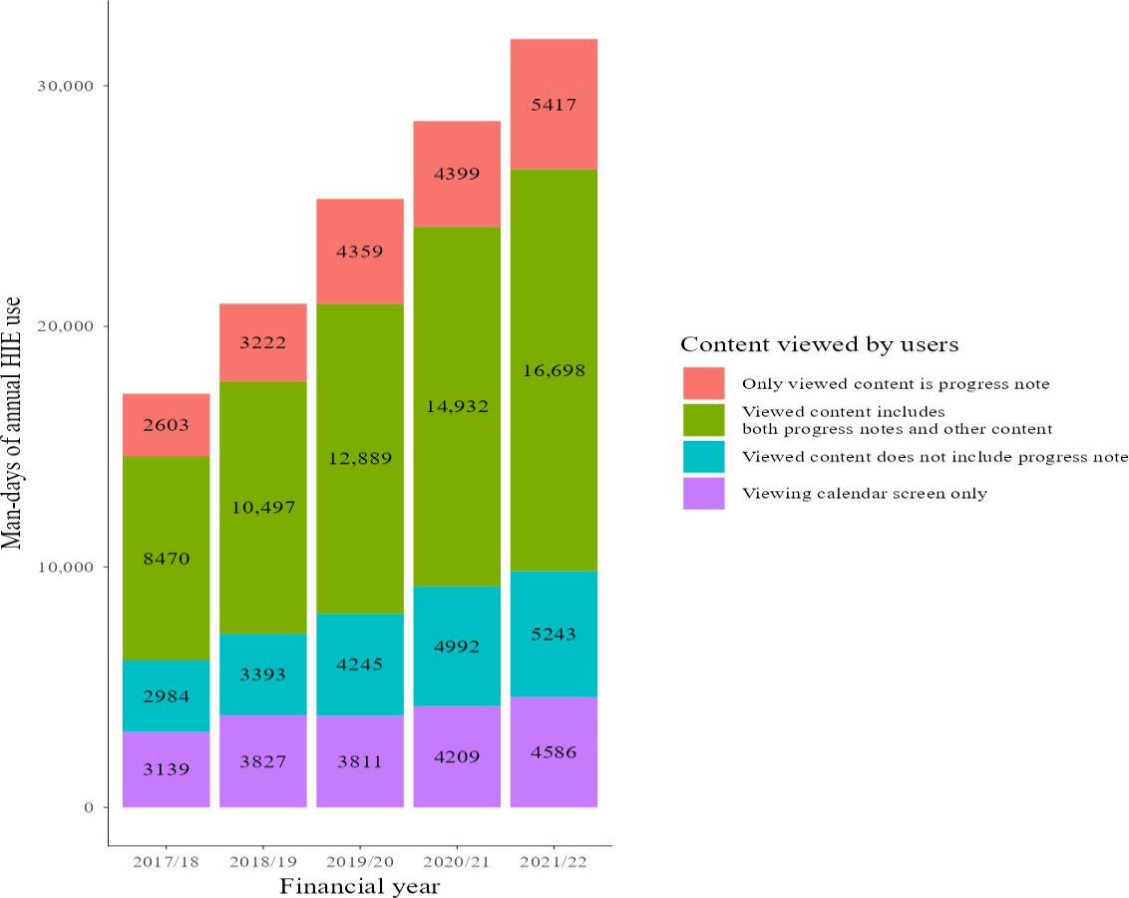
Top 5 content views by man-days on Choukai Net. HIE: health information exchange.

**Figure 3.**
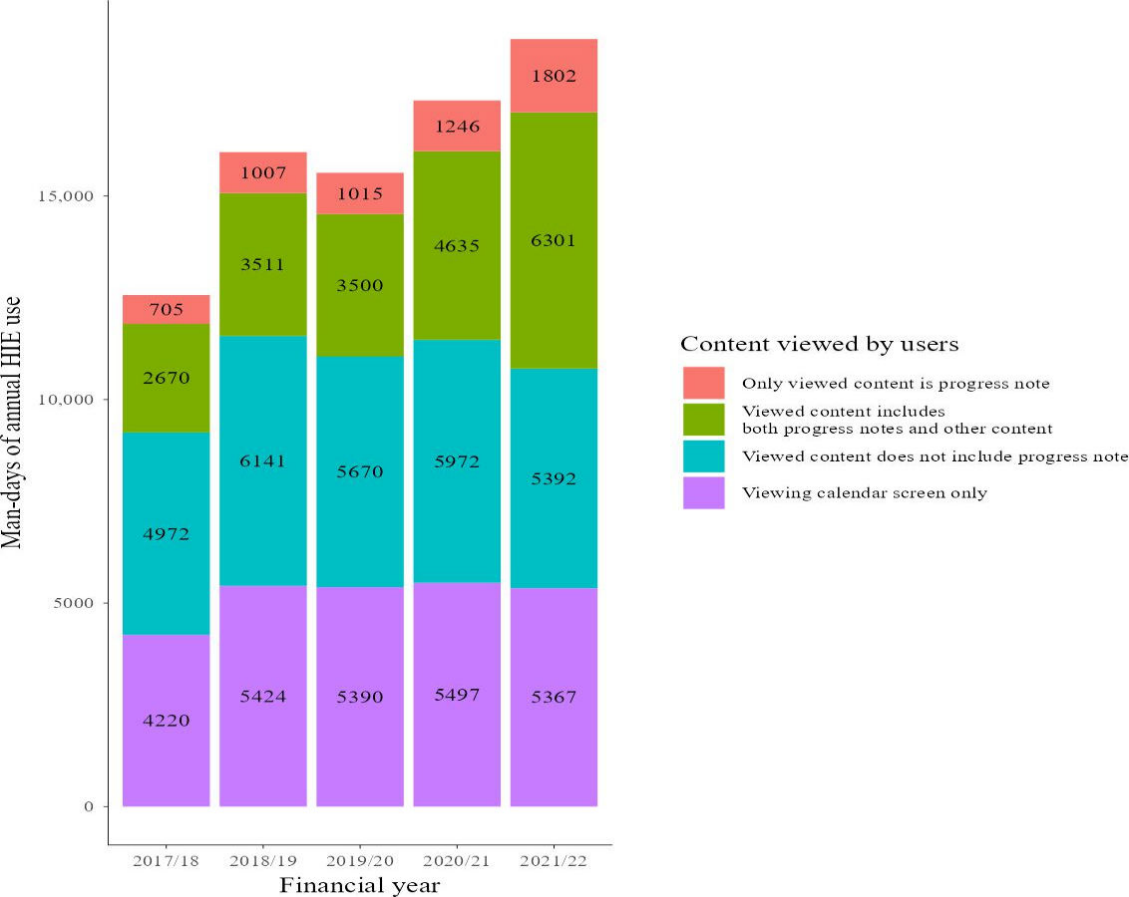
Top 5 content views by man-days on PicaPicaLink. HIE: health information exchange.

We present the top 5 content views by man-days over the total data analysis period in Figures2  3. Figure 2 presents the man-days on Choukai Net, and Figure 3 presents the man-days for PicaPicaLink. Man-days and the view rates including other types of content views, not just the top 5, are listed in Tables S4 and S5 in Multimedia Appendix 3. These supplementary tables include content with a total view rate of 1% or more.

#### Man-Days and View Rate for Content and Output Field by Institution Type in FY 2021/22

The view rates for each output field and content by institution type are listed in Tables5  6. Table 5 presents the view rate for Choukai Net, and Table 6 presents the view rate for PicaPicaLink. These tables include content with a total view rate of 1% or more in FY 2021/22.

**Table 5.** Man-days and view rate for each output field and content by institution type in financial year 2021/22 on Choukai Net.

Output field and content		Hospital (total man-days=18,356), n (%)	Medical clinic (total man-days=6309), n (%)	Dental clinic (total man-days=19), n (%)	Pharmacy (total man-days=568), n (%)	Visiting nursing station (total man-days=6052), n (%)	Nursing facility (total man-days=5277), n (%)	Total, n (%)
**Note**								
	Total contents	7187 (51.9)	4824 (76.5)	6 (31.6)	463 (81.5)	5601 (92.5)	4857 (92)	22,933 (71.8)
	Progress note	6598 (47.6)	4715 (74.7)	6 (31.6)	457 (80.5)	5553 (91.8)	4784 (90.7)	22,108 (69.3)
	Patient summary	3743 (27)	2132 (33.8)	3 (15.8)	121 (21.3)	1015 (16.8)	1162 (22)	8173 (25.6)
	Disease name	279 (2)	123 (1.9)	0 (0)	0 (0)	87 (1.4)	519 (9.8)	1008 (3.2)
	Allergy	183 (1.3)	56 (0.9)	0 (0)	0 (0)	164 (2.7)	596 (11.3)	999 (3.1)
	Nursing record	156 (1.1)	150 (2.4)	0 (0)	0 (0)	130 (2.1)	185 (3.5)	621 (1.9)
**Reports**								
	Total contents	2969 (21.4)	3269 (51.8)	1 (5.3)	107 (18.8)	1399 (23.1)	934 (17.7)	8676 (27.2)
	Patient referral document	2091 (15.1)	1976 (31.3)	0 (0)	51 (9)	668 (11)	472 (8.9)	5256 (16.5)
	Radiology report	1275 (9.2)	2051 (32.5)	1 (5.3)	46 (8.1)	476 (7.9)	365 (6.9)	4213 (13.2)
	Discharge summary	514 (3.7)	589 (9.3)	0 (0)	40 (7)	270 (4.5)	156 (3)	1569 (4.9)
	Nursing summary	468 (3.4)	268 (4.2)	0 (0)	25 (4.4)	518 (8.6)	169 (3.2)	1448 (4.5)
Prescription		1901 (13.7)	1596 (25.3)	3 (15.8)	318 (56)	2071 (34.2)	1297 (24.6)	7186 (22.5)
**Test results**								
	Total content	1403 (10.1)	2113 (33.5)	1 (5.3)	174 (30.6)	1376 (22.7)	1018 (19.3)	6085 (19.1)
	Laboratory test result	1329 (9.6)	2095 (33.2)	1 (5.3)	173 (30.5)	1342 (22.2)	981 (18.6)	5921 (18.6)
	Bacterial examination test result	280 (2)	218 (3.5)	0 (0)	12 (2.1)	118 (1.9)	105 (2)	733 (2.3)
DICOM^a^ image list		2712 (19.6)	1239 (19.6)	2 (10.5)	0 (0)	294 (4.9)	159 (3)	4406 (13.8)
**Image test order**								
	Total content	1500 (10.8)	1149 (18.2)	1 (5.3)	56 (9.9)	661 (10.9)	685 (13)	4051 (12.7)
	Radiology imaging order	1335 (9.6)	936 (14.8)	1 (5.3)	46 (8.1)	600 (9.9)	571 (10.8)	3489 (10.9)
	Physiological test order	437 (3.2)	431 (6.8)	0 (0)	11 (1.9)	89 (1.5)	184 (3.5)	1152 (3.6)
	Endoscopy order	84 (0.6)	159 (2.5)	0 (0)	2 (0.4)	33 (0.5)	49 (0.9)	327 (1)
Injection		601 (4.3)	456 (7.2)	1 (5.3)	72 (12.7)	976 (16.1)	617 (11.7)	2723 (8.5)
Summary view		298 (2.2)	230 (3.6)	1 (5.3)	21 (3.7)	70 (1.2)	110 (2.1)	730 (2.3)
Chart display		174 (1.3)	107 (1.7)	0 (0)	1 (0.2)	192 (3.2)	96 (1.8)	568 (1.8)
File list		237 (1.7)	70 (1.1)	0 (0)	0 (0)	5 (0.1)	1 (0)	313 (1)

a DICOM: Digital Imaging and Communications in Medicine.

**Table 6.** Man-days and view rate for each output field and content by institution type in financial year 2021/22 on PicaPicaLink.

Output field and content		Hospital (total man-days=8878), n (%)	Medical clinic (total man-days=5434), n (%)	Pharmacy (total man-days=1941), n (%)	Visiting nursing station (total man-days=193), n (%)	Nursing facility (total man-days=2234), n (%)	Total, n (%)
**Note**							
	Total content	2417 (27.2)	3336 (61.4)	1064 (54.8)	128 (66.3)	1208 (54.1)	8153 (43.6)
	Progress note	2340 (26.4)	3301 (60.7)	1064 (54.8)	126 (65.3)	1201 (53.8)	8032 (43)
	Nursing record	287 (3.2)	197 (3.6)	0 (0)	43 (22.3)	112 (5)	639 (3.4)
	Disease name	187 (2.1)	246 (4.5)	6 (0.3)	0 (0)	49 (2.2)	488 (2.6)
**Reports**							
	Total content	1751 (19.7)	2896 (53.3)	308 (15.9)	39 (20.2)	1011 (45.3)	6005 (32.1)
	Radiology report	1007 (11.3)	1939 (35.7)	104 (5.4)	25 (13)	621 (27.8)	3696 (19.8)
	Patient referral document	848 (9.6)	1495 (27.5)	173 (8.9)	9 (4.7)	447 (20)	2972 (15.9)
	Discharge summary	243 (2.7)	468 (8.6)	113 (5.8)	7 (3.6)	211 (9.4)	1042 (5.6)
	Nursing summary	242 (2.7)	261 (4.8)	19 (1)	5 (2.6)	281 (12.6)	808 (4.3)
	Surgical record	122 (1.4)	143 (2.6)	2 (0.1)	0 (0)	0 (0)	267 (1.4)
	Other documents	114 (1.3)	88 (1.6)	0 (0)	0 (0)	3 (0.1)	205 (1.1)
**Test results**							
	Total content	1397 (15.7)	2342 (43.1)	1041 (53.6)	90 (46.6)	827 (37)	5697 (30.5)
	Laboratory test result	1383 (15.6)	2327 (42.8)	1038 (53.5)	90 (46.6)	826 (37)	5664 (30.3)
	Bacterial examination test result	161 (1.8)	105 (1.9)	26 (1.3)	0 (0)	9 (0.4)	301 (1.6)
Prescription		1115 (12.6)	1466 (27)	728 (37.5)	113 (58.5)	926 (41.5)	4348 (23.3)
**Image test order**							
	Total content	718 (8.1)	1226 (22.6)	274 (14.1)	6 (3.1)	406 (18.2)	2630 (14.1)
	Radiology imaging order	686 (7.7)	1193 (22)	267 (13.8)	6 (3.1)	368 (16.5)	2520 (13.5)
	Physiological test order	110 (1.2)	146 (2.7)	36 (1.9)	1 (0.5)	111 (5)	404 (2.2)
**Injection**							
	Total content	611 (6.9)	791 (14.6)	623 (32.1)	55 (28.5)	406 (18.2)	2486 (13.3)
	Injection order	536 (6)	582 (10.7)	543 (28)	49 (25.4)	405 (18.1)	2115 (11.3)
	Injection	127 (1.4)	314 (5.8)	145 (7.5)	18 (9.3)	14 (0.6)	618 (3.3)
DICOM^a^ image list		1196 (13.5)	1133 (20.9)	0 (0)	2 (1)	91 (4.1)	2422 (13)
**Summary view**							
	Total content	221 (2.5)	169 (3.1)	237 (12.2)	21 (10.9)	41 (1.8)	689 (3.7)
	Examination summary view	158 (1.8)	137 (2.5)	180 (9.3)	18 (9.3)	12 (0.5)	505 (2.7)
	Medication summary view	87 (1)	51 (0.9)	92 (4.7)	6 (3.1)	33 (1.5)	269 (1.4)
Chart display		34 (0.4)	3 (0.1)	7 (0.4)	0 (0)	136 (6.1)	180 (1)

a DICOM: Digital Imaging and Communications in Medicine.

Progress notes are the most frequently viewed content at every institution type on both HIEs. When comparing the view rate of progress notes by institution type, visiting nursing stations had the highest rates, with 91.8% for Choukai Net and 65.3% for PicaPicaLink. On the other hand, the view rate of the Digital Imaging and Communications in Medicine (DICOM) image list was relatively low for most institution types. In both HIEs, the view rate of the DICOM image list was less than 5% in pharmacies, visiting nursing stations, and nursing facilities. The view rate for radiology reports was higher than for DICOM image lists in all these institution types. Even in medical clinics, the view rate for DICOM image lists was lower than the view ratio for radiology reports, indicating that many medical workers view the radiology reports written by radiologists rather than viewing the DICOM images. Hospitals were the only institution category where the view rate for DICOM image lists was higher than for radiology reports.

#### Man-Days of Viewing Content Out of the Total Man-Days of Viewing the Calendar and the Breakdown of Progress Note Views

**Figure 4.**
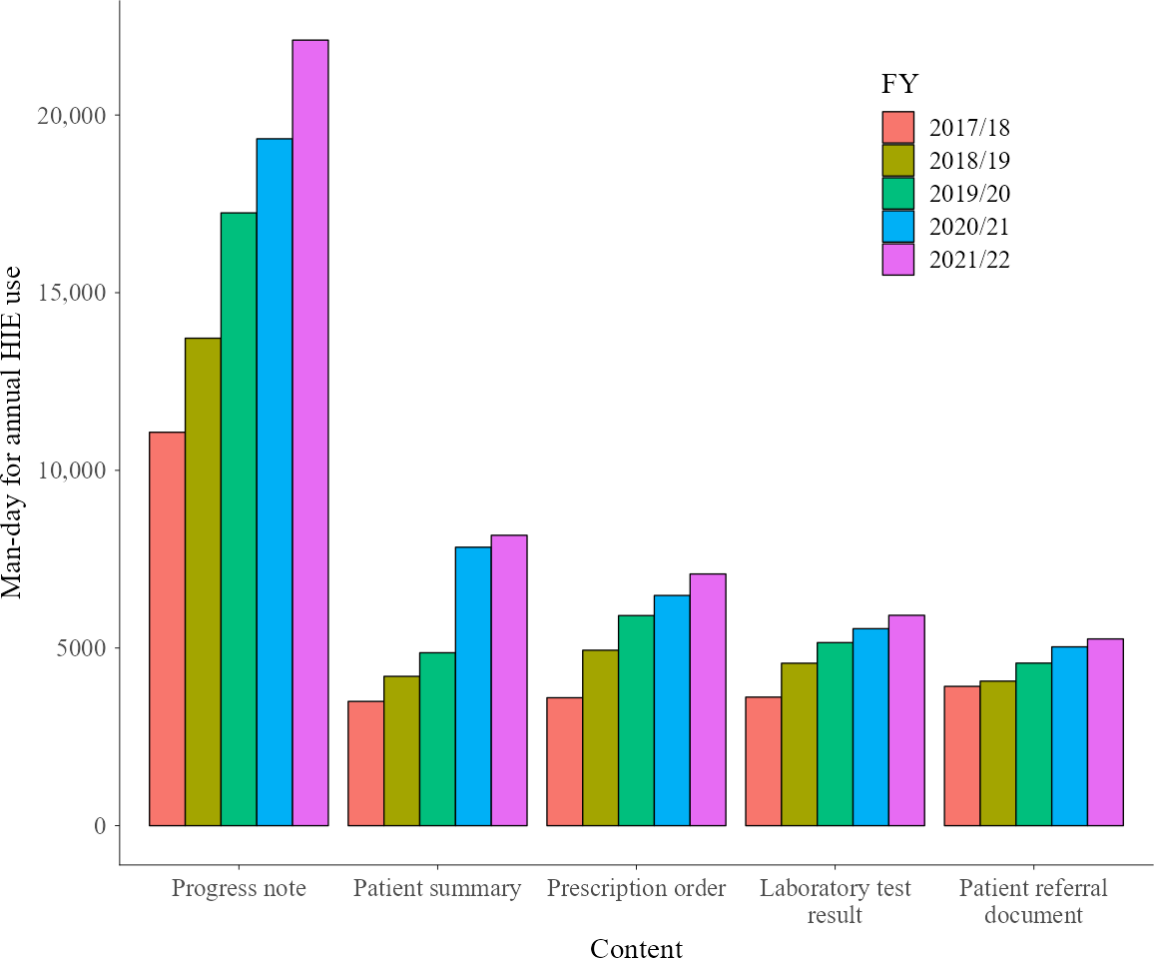
Content viewed by users when using Choukai Net. FY: financial year; HIE: health information exchange.

**Figure 5.**
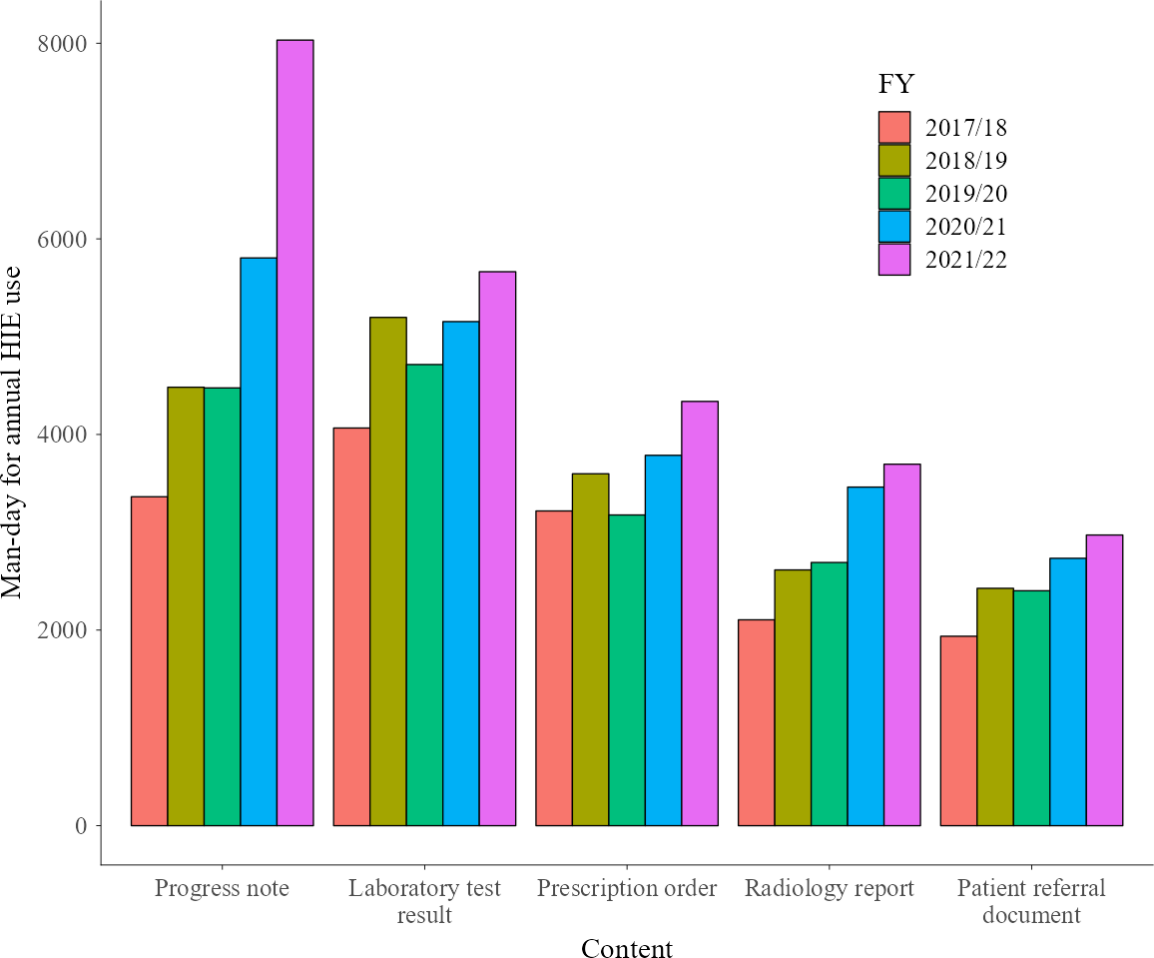
Content viewed by users when using PicaPicaLink. Views of Sagan-Path Net are not included in the statistics. FY: financial year; HIE: health information exchange.

The following presents the results of an analysis of content views and calendar views, and the percentage of progress note views among content views. The aggregated results are shown as bar graphs, with Choukai Net illustrated in Figure 4 and PicaPicaLink in Figure 5. On Choukai Net, the number of man-days in which progress notes were the only content viewed accounted for 17% (5417/31,944) of the total in FY 2021/22, a rate not significantly different from 15.1% (2603/17,196) in FY 2017/18. This may be because the medical institutions that disclose progress notes have remained unchanged from FY 2017/18 to FY 2021/22 (Table S1 in Multimedia Appendix 1).

In contrast, on PicaPicaLink, the disclosure of progress notes was implemented in stages over 5 years (Table S2 in Multimedia Appendix 1). At the beginning of FY 2017/18, only 2 of the 13 DDIs were disclosing progress notes. Over the 5 years analyzed, the DDIs of PicaPicaLink proceeded to disclose progress notes, and by March 2022, 8 of 15 DDIs had done so. Therefore, the proportion of viewing only progress notes on PicaPicaLink also increased from 5.6% (705/12,567) in FY 2017/18 to 9.6% (1802/18,862) in FY 2021/22.

#### Man-Days of Views of Content Corresponding to 3D6I and Non-3D6I Content

In Table 7, we present the man-days of total views, views of content corresponding to 3D6I, and views of content not corresponding to 3D6I by FY. The view rate of content corresponding to 3D6I was below 50%. On the other hand, the view rate of content not included in the 3D6I consistently exceeded 50%.

**Table 7.** Man-days of total views, views of content corresponding to 3D6I, and views of content not corresponding to 3D6I by financial year.^a^

Health information exchange and financial year		Total number of man-days	3D6I, number of man-days (%)	Other information, number of man-days (%)
**Choukai Net**				
	2017/18	17,187	7806 (45.4)	13,351 (77.7)
	2018/19	20,910	9666 (46.2)	16,252 (77.7)
	2019/20	25,262	11,784 (46.6)	20,394 (80.7)
	2020/21	28,514	13,022 (45.7)	23,117 (81.1)
	2021/22	31,918	14,221 (44.6)	26,120 (81.8)
	Total	123,791	56,499 (45.6)	99,234 (80.2)
**PicaPicaLink**				
	2017/18	12,447	6138 (49.3)	6561 (52.7)
	2018/19	15,953	7722 (48.4)	8504 (53.3)
	2019/20	15,396	7093 (46)	8286 (53.8)
	2020/21	17,136	7998 (46.7)	9975 (58.2)
	2021/22	18,680	9021 (48.3)	11,726 (62.8)
	Total	79,612	37,972 (47.7)	45,052 (56.6)

a 3D6I: 3 documents and 6 types of information.

## Discussion

### Principal Findings

As mentioned in the Results section, the use of Choukai Net in hospitals and visiting nursing stations is more active than in previous studies (Tables2  3). These findings suggest that the frequency of HIE use in Japan is not necessarily low and that certain factors may contribute to increased usage. To clarify these reasons, a survey of individual facilities using HIEs is needed rather than a survey of overall HIE usage. Although it is difficult to attribute the cause of active use to general statistical indicators of the region, low population density may have increased the need for medical coordination between institutions (Table 1). On the other hand, the population density of Saga Prefecture, where PicaPicaLink is operated, is roughly the same as the national average.

In both HIEs analyzed, the most viewed data were progress notes (Figures2  3). In FY 2021/22, 17% of Choukai Net usage and 9.6% of PicaPicaLink usage involved referencing only progress notes (Figures4  5). The overall view rate for progress notes was 67.4% for Choukai Net and 32.9% for PicaPicaLink. One reason for the more than 2-fold difference in view rate between the 2 HIEs is that each DDI has different progress note disclosure policies. On Choukai Net, 5 of the 7 DDIs had been disclosing progress notes since April 2017, and none changed their disclosure policies until March 2022 (Table S1 in Multimedia Appendix 1). In contrast, only 2 of the 13 DDIs of PicaPicaLink had disclosed progress notes as of April 2017 (Table S2 in Multimedia Appendix 1). As a result, the view rate of progress notes in FY 2017/18 was only 27%. However, in FY 2021/22, when 8 of 15 DDIs were disclosing progress notes, the view rate increased to 43%. The differences between the 2 HIEs and increase in progress note viewing rate on PicaPicaLink suggest that DDI data disclosure policies influence health care workers’ data viewing behavior in HIEs. Previous studies have not necessarily examined the specific types of data disclosed by each DDI in HIEs, but this aspect is crucial for understanding HIE usage.

The results of these analyses do not necessarily align with those of previous studies. Laboratory results, medication data, and radiology data have been reported as the most frequently accessed types of data in HIEs [24-26]. This difference is partly because the number of views for progress notes was not aggregated in previous research. A recent study conducted in 2024 on Mame-Net, one of Japan’s HIEs, found that medical records were the most frequently viewed, which is consistent with the findings of this study [27].

When comparing the view rate of progress notes by institution type, visiting nursing stations had the highest rates, with 91.8% for Choukai Net and 65.3% for PicaPicaLink (Tables4  5). The distribution of occupations using HIEs is strongly influenced by institution type, and most users of HIE at visiting nursing stations are nurses (Table 4). It is understandable that the progress notes written by doctors are important for nurses who work based on doctors’ instructions [28]. A similar trend has been observed in pharmacies, where pharmacies dispense medicines based on a doctor’s prescription and therefore must check the doctor’s notes to validate the prescription. However, the viewing rate of patient summaries, which are also written by physicians, is low across all institution types. Patient summaries or patient referral documents do not necessarily include detailed medical histories. Additionally, information is generally updated more frequently in progress notes. These factors may explain why progress notes are viewed more frequently. Further research, including qualitative studies, is needed to explore the benefits of disclosing progress notes and how they can be used in DVI.

Despite relatively high demand compared to other types of data, progress note disclosure is uncommon in Japanese HIEs. A survey conducted on HIEs in 2023 found that the disclosure rate of progress notes written by doctors was only 38.6% (81/210) [29]. The most common reason for not disclosing progress notes, cited by 48.6% (35/72) of HIEs, was the “lack of consent from DDIs to share progress notes.” The fourth most common reason, cited by 15.3% (11/72) of HIEs, was that “it may limit doctors’ entries in progress notes.” These results suggest that concerns about violating patient and doctor privacy, owing to the sensitive information contained in progress notes, prevent their disclosure. On the other hand, the second most common reason, cited by 41.7% (30/72) of HIEs, was that “the system did not have a progress note sharing function”. The third most common reason, given by 16.7% (12/72) of HIEs, was that “management organizations consider it unnecessary to disclose progress notes.” These results indicate that the system would need modification to enable progress note sharing and this is not currently considered worth the cost in these HIEs. Past surveys have shown that the benefits of disclosing progress notes and reports include the ability to quickly share detailed medical information and improvements in the quality of progress notes [29]. However, these descriptions remain abstract, and the benefits are not quantified. Further research is needed to quantify the benefits of disclosure and evaluate whether they justify the additional costs and privacy risks. Moreover, efforts must be made to maximize the benefits relative to the costs of disclosure while minimizing privacy concerns.

The results of this study raise questions about the effectiveness of CLINS. Progress notes and radiology reports are not included in the 3D6I that CLINS plans to share. The discharge summary, a physician’s record written in natural language, is included in 3D6I, but its view rate was less than 10% in this analysis. In the HIEs analyzed in this study, the view rate of 3D6I was less than 50%, which is lower than the view rate of data not included in 3D6I (Table 7). However, except for patient referral documents and discharge summaries, data shared via CLINS can be accessed by both patients and medical professionals. In contrast, most regional HIEs in Japan, including the 2 HIEs studied here, do not allow patients to view shared data using the HIE. Past surveys have shown that only 6.3% (14/224) of HIEs in Japan offer personal health record functionality [29]. Future evaluations of CLINS and regional HIEs should assess not only the types of data shared but also whether or not it is shared with patients.

It is necessary to consider whether the usefulness of the HIE analyzed in this study is worth the cost. There was no publicly available data on the costs of PicaPicaLink, making it difficult to estimate the cost-benefits. Regarding Choukai Net, some basic operational figures were presented at an academic conference in 2018 [30]. Excluding implementation cost, the annual maintenance costs are 3.89 million yen (US $27,020) for total data center usage, and 1.03 million yen (US $7020) for the HIE Management Council. The cost of replacing the systems at the 3 hospitals is 12.1 million yen (US $83,180). Assuming that the average system replacement cost for 1 facility is one-third of this amount, the cost of replacing the systems at all 7 DDI facilities can be roughly estimated to be 28.2 million yen (US $193,850). Assuming that system replacement occurs once every 5 years, the annual reserve required for system replacement of all DDIs of Choukai Net is 5.64 million yen (US $38,760). Therefore, the annual cost of data center usage fees, council operating fees, and annual reverse funds for system replacement of DDIs is about 10.6 million yen (US $72,850) per year. If we divide these annual costs by the number of man-days of Choukai Net usage in FY 2021/22, 31,918 person-days, we get an approximate cost of 331 yen (US $2.28) per 1 man-day of HIE use. Further research is needed to determine whether the health care workers’ benefits per man-day of use are commensurate with these costs. On the other hand, from the perspective of benefits to patients, a randomized controlled trial on the benefits of HIEs to patients has been conducted in Japan [31]. However, the results were not necessarily clear, and analysis of HIE use or viewed data was not included. Further research is also needed to assess the benefits of HIEs for patients, including the associated costs and actual utilization.

As detailed in the “Patient consent” section under “Methods,” patients must fill out a paper consent form each time to increase the number of sources or recipients of the patient medical information shared. This method takes privacy into consideration as patients have some control over whether or not their medical data are disclosed to specific medical institutions. This consent method is common in Japanese HIEs. Previous studies have shown that 87.8% (195/222) of HIEs in Japan require patients to complete a consent form when they first join the HIE. In addition, 58.5% (103/176) of HIEs require patients to complete additional consent forms when transferring patient medical information between new facilities [3]. However, the MHLW has notified that patient completion of a consent form is not necessarily required for them to participate in an HIE [32]. In addition, previous studies and reports have pointed out that opt-in consent policies not only impose an administrative burden on hospital medical staff, but also have the potential to lower consent rates and hinder the use of HIEs [33-35]. A future challenge for regional HIEs in Japan is how to reduce the effort of medical workers to obtain the consent required to use HIEs while respecting patient privacy. Recent research suggests that a hybrid combination of opt-in and opt-out policies can balance privacy considerations with the quality of consent [35]. As another approach, previous systematic reviews have noted that patients’ perceptions of the benefits of HIEs may alleviate privacy concerns [36]. Another study also suggested that when patients are aware of an HIE’s privacy policy and sharing procedures, and have trust in the HIE, they may be more likely to disclose their health information [37]. As we continue to study the benefits of HIEs and share these findings, it is possible that patients become less wary of HIEs and require simpler consent.

### Limitations

This study had some limitations. The most significant limitation was that we analyzed data from only 2 HIEs. For example, the view rate of each item in HIEs that do not publish progress notes may differ significantly from the results of this study. As we have not been able to obtain audit log data, the detailed usage status of Sagan-Pass Net is unknown. In addition, because this study was a quantitative analysis of audit logs, no questionnaires or interview surveys were conducted to directly ask medical workers about the factors influencing differences in utilization and content-viewing rates. A combination of audit log analysis and qualitative research will be necessary to identify in what situations medical data are being accessed during daily work and for what purposes.

### Conclusions

In both HIEs analyzed in this study, progress notes were the most viewed content. As more health care organizations disclose the progress notes they manage to HIEs, their view rates are likely to increase. The cost-benefit of disclosing progress notes to HIEs remains unclear, and both health care providers and patients are concerned about privacy risks. Future research is needed to quantify and maximize the benefits of disclosure while minimizing privacy risks.

## Supplementary material

10.2196/65575 Multimedia Appendix 1 List of institutions that disclose electronic medical record data to health information exchanges and the disclosed data.

10.2196/65575 Multimedia Appendix 2 Results of the content sort.

10.2196/65575 Multimedia Appendix 3 Man-days and view rate of each output field and content by financial year.

## Abbreviations

3D6I: 3 documents and 6 types of information
CLINS: Clinical Information Sharing
DDI: data disclosure institution
DICOM: Digital Imaging and Communications in Medicine
DVI: data viewing institution
EMS: emergency medical service
FY: financial year
HIE: health information exchange
MHLW: Ministry of Health, Labour and Welfare
NMF: name matching feature
PDDSF: patient demographic data synchronization feature
