# Supplementary material for: Health Information Exchange Usage in Japan: Content Analysis of Audit Logs
Source: JMIR Med Inform. 2025 May 27;13:e65575. doi: 10.2196/65575 (PMC12133072; doi:10.2196/65575)
Supplement: Multimedia Appendix 2 [file medinform-v13-e65575-s002.docx]

Table S3. Result of the content sort.

| output field | content | 3D6I ^a^ |
| --- | --- | --- |
| Waveform data list | electrocardiogram waveform | 0 |
| Injection | Injection (performing) | 0 |
| Injection | Injection (order) | 0 |
| Meal | Meal order | 0 |
| Prescription | dispensing information (Pharmacy） | 1 |
| Prescription | prescription order | 1 |
| Test results | laboratory test result | 1 |
| Test results | bacterial examination test result | 0 |
| Medical check-up | Medical check-up | 1 |
| Image list | DICOM image | 0 |
| Image test order | physiological test order | 0 |
| Image test order | endoscopy order | 0 |
| Image test order | radiology imaging order | 0 |
| Reports | patient referral document | 1 |
| Reports | discharge summary | 1 |
| Reports | surgical record | 0 |
| Reports | radiology report | 0 |
| Reports | nursing summary | 0 |
| Reports | other documents | 0 |
| Face sheet | vital sign | 0 |
| Face sheet | social history | 0 |
| Face sheet | progress record | 0 |
| Face sheet | medical history | 0 |
| Face sheet | blood type | 0 |
| Face sheet | declaration of intention | 0 |
| Face sheet | medication information | 0 |
| Face sheet | medical note | 0 |
| Face sheet | test result | 0 |
| Face sheet | providing information | 0 |
| Face sheet | physical measurement | 0 |
| Face sheet | family history | 0 |
| Face sheet | consultation information | 0 |
| Face sheet | contact address | 0 |
| Face sheet | list of authors | 0 |
| Face sheet | allergy | 0 |
| Face sheet | activities of daily living | 0 |
| Face sheet | ACP | 0 |
| File list | File list | 0 |
| Note | infectious disease information | 1 |
| Note | allergy | 1 |
| Note | disease name | 1 |
| Note | progress note | 0 |
| Note | nursing record | 0 |
| Note | patient summary | 0 |
| Note | other records | 0 |
| Chart display | vital sign | 0 |
| Chart display | pedometer chart | 0 |
| Chart display | exercise intensity chart | 0 |
| Summary view | summary view (prescription) | 1 |
| Summary view | summary view (prescription and injection) | 1 |
| Summary view | summary view (time series of test results) | 1 |
| Summary view | summary view (test results） | 1 |
| Summary view | summary view (prescription and test results) | 1 |

a Content that corresponds to 3D6I is indicated as 1, and content that does not correspond is indicated as 0.
